# Adolescent-Parent Attachment and Externalizing Behavior: The Mediating Role of Individual and Social Factors

**DOI:** 10.1007/s10802-015-9999-5

**Published:** 2015-03-15

**Authors:** Sanne L. A. de Vries, Machteld Hoeve, Geert Jan J. M. Stams, Jessica J. Asscher

**Affiliations:** 1 Research Institute of Child Development and Education, University of Amsterdam, Nieuwe Achtergracht 127, 1018 WS Amsterdam, The Netherlands; 2 Research Institute of Child Development and Education, University of Amsterdam, P.O. Box 15780, 1001 NG Amsterdam, The Netherlands

**Keywords:** Attachment, Externalizing behavior, Self-esteem, Cognitive distortions, Deviant peers, Parental monitoring

## Abstract

The aim of this study was to test whether the associations between adolescent-parent attachment and externalizing problem behavior of adolescents were mediated by adolescent cognitive distortions, self-esteem, parental monitoring and association with deviant peers. A total of 102 adolescents (71 % male; aged 12–19 years) at risk for developing delinquent behaviors reported on attachment, parental monitoring, aggressive and delinquent behavior and peers. Mediation effects were tested by using structural equation modeling. Different pathways were found depending on the type of externalizing behavior. The association between attachment and direct and indirect aggressive behavior was mediated by cognitive distortions. The relation between attachment and delinquency was mediated by deviant peers and parental monitoring. We argue that clinical practice should focus on the attachment relationship between adolescent and parents in order to positively affect risk and protective factors for adolescents’ aggressive and delinquent behavior.

**Electronic supplementary material:**

The online version of this article (doi:10.1007/s10802-015-9999-5) contains supplementary material, which is available to authorized users.

Meta-analytic studies by Fearon et al. (2010) and Hoeve et al. (2012) have shown that adolescents’ attachment to their parents is associated with concurrent and later aggression and delinquency. Few studies, however, have examined the mechanisms that could explain the association between attachment and these externalizing behaviors. Adolescents have developed mental representations of self and others in attachment relationships with their parents that shape both individual and social functioning. In the present study, we empirically test whether risk and protective factors of individual functioning (i.e., cognitive distortions and self-esteem) and social functioning (affiliations with deviant peers, and parental monitoring through adolescents’ self-disclosure) mediate the association between attachment and externalizing behavior (i.e., aggression and delinquency).

## Mediation Through Cognitive Distortions

Children internalize both secure and insecure patterns of their relationships with caregivers as mental representations or internal working models of attachment (Dykas and Cassidy 2011; Pietromonaco and Barrett 2000), which influences the way children interact with their environment (Bowlby 1973). Individuals with secure internal working models process a broad range of positive and negative attachment-relevant experiences, and their mental schemas represent a coherent integration and organization of these experiences (Bowlby 1969; Pietromonaco and Barrett 2000). Security of attachment facilitates cognitive abilities (e.g., memory and comprehension) and social understanding (Fonagy and Target 1997). Insecure attachment organizations are characterized by the defensive exclusion of information or inability to integrate different types of information about attachment experiences, which may lead to distorted communications and negative expectations of self in relationship with others (Cassidy et al. 1996; Dodge 1993; Shumaker et al. 2009). Children internalize negative experiences with their parents as insecure internal working models of attachment (Blatt and Homann 1992), which have been linked to poor mentalizing abilities (Fonagy and Target 1997), hampering perspective taking and making adolescents vulnerable to egocentric bias and self-serving cognitive distortions, defined as “inaccurate attitudes, thoughts or beliefs concerning own or others’ behavior” (Gibbs et al. 1995, p. 165). These types of distortions buffer the self from blame or negative self-concept, which reinforces aggression or other forms of antisocial behavior (Barriga et al. 2000). Helmond et al. (2014) showed in their meta-analysis that cognitive distortions are moderately associated with both aggression and delinquency.

## Mediation Through Self-Esteem

Attachment to parents has been shown to be associated with adolescents’ self-esteem (e.g., Armsden and Greenberg 1987; Gamble and Roberts 2005; Lee and Hankin 2009; Noom et al. 1999; Papini and Roggman 1992; Paterson et al. 1995; Roberts et al. 1996), defined as self-judgments of personal worth and global feelings of competence and self-acceptance (Rosenberg 1965). Through attachment relationships children develop a working model of the self, which consists of generalized perceptions of competence and self-esteem (Greenberg et al. 1993). Children who perceive their parents as being responsive and available are likely to internalize a sense of their own self-worth and expect that others will attend to their needs (see Gerlsma et al. 1996). In contrast, children with insecure working models of attachment view others as untrustworthy or unavailable, which in turn leads to a lack of confidence in self and others (Gamble and Roberts 2005; Gomez and McLaren 2007).

The link between low self-esteem and externalizing problems has been well established in empirical research (e.g., Donnellan et al. 2005; Trzesniewski et al. 2006), and may be explained in different ways. According to Rosenberg (1965), low self-esteem weakens ties with society, and low engagement with society in turn decreases conformity to social norms and increases delinquency (Hirschi 1969). It has also been suggested that adolescents with low self-esteem show various forms of antisocial behavior, including aggression, as a way of enhancing their self-worth (Kaplan 1980).

## Mediation Through Parental Monitoring

Several studies have suggested that attachment is related to self-disclosure (Keelan et al. 1998; Pistole 1993), that is, youths’ tendencies to provide unsolicited information (Kerr and Stattin 2000). Individuals with secure attachments experience a sense of worthiness, which contributes to more engagement in self-disclosure (Keelan et al. 1998). Self-disclosure involves a significant amount of trust. Trust is related to the understanding one has of others’ likely responses to personal vulnerability, also referred to as an internal working model of relationships with others (Mount 2005). In particular, trust in others has been linked to the amount of information self-disclosed to another (Levin and Gergen 1969; Pearce 1974). A positive and trusting relationship between parents and adolescents creates an open way of communication about adolescents’ daily activities, thoughts and feelings (Deković et al. 2004). Kerr and Stattin (2000) found that adolescent disclosure contributes to greater parental knowledge of adolescents’ whereabouts.

In contrast, insecure representations of attachment with parents could lead to less self-disclosure and parental monitoring (Branstetter et al. 2009). Attachment insecurity has been found to predict greater reluctance of adolescents to provide their parents information on their whereabouts and activities (Kerns et al. 2001; Sampson and Laub 1994). Insecurely attached adolescents tend to spend less leisure time in parental company and are more attracted by unsupervised peer settings (Kerr and Stattin 2000). In addition, insecurity may lead to externalizing behavior by causing hostility toward parents or efforts to minimize conscious attention directed toward parents, either of which may reduce behavioral parental control (Allen et al. 1998). Several studies have concluded that low levels of parental monitoring and lack of knowledge are associated with adolescents’ involvement in a range of antisocial and delinquent behaviors (see Crouter and Head 2002; Dishion and McMahon 1998; Patterson 1986). Inconsistent and erratic supervision by parents promotes deviant attitudes and behaviors in their children (Akers 2000).

## Mediation Through Deviant Peers

Empirical support exists for the association between attachment and peer affiliations (Benson et al. 2006; Warr 1993). Representations of relationships with parents shape a child’s core strategy of regulating his/her emotions, thoughts and behaviors in close relationships, such as friendships (Bowlby 1973). According to Hirschi’s (1969) social control theory adolescents who are strongly attached to their parents may be less influenced by deviant peers. These adolescents are more prone to seek out nondelinquent peers to avoid parental disapproval or because their parents actively regulate their children’s friendships to avoid undesirable peers (Warr 1993).

Youth with insecure attachment relationships have poorer levels of social competence and more negative friendships (Shulman et al. 1994). Negative interactions with parents interfere with effective functioning of a secure base from which adolescents can form friendships, which hampers adolescents’ ability to establish positive friendships (Shomaker and Furman 2009). Moreover, parental rejection or absence of close bonds with parents leads to an adolescent’s rejection of commitment to conventional values. Subsequently, adolescents rejecting conventional values are more likely to associate with peers who support unconventional standards. In turn, these peers act as role models in learning or reinforcing delinquent behavior that adolescents tend to imitate (Akers 2000). Many studies have considered that affiliations with antisocial and deviant peers are related to various problematic outcomes during adolescence, such as high levels of aggression (Benson and Buehler 2012; Capaldi et al. 2001), police arrests (Patterson et al. 2000), and other forms of antisocial behavior (Ardelt and Day 2002; Reitz et al. 2006; Vitaro et al. 2000).

## Etiology of Aggressive and Delinquent Behavior

Currently, several studies and classification systems for child and adolescent psychopathology distinguish between aggressive and delinquent behavior, because these two forms of externalizing behavior seem to differ in several aspects. Firstly, several studies showed that aggressive and delinquent behavior are distinct at the etiologic level. Although the interplay of genetics and the environment influences both types of antisocial behavior (aggression and delinquency), genetic influences were suggested to be greater for aggressive antisocial behavior than for nonaggressive antisocial behavior (Eley et al. 2003). Moreover, Tackett et al. (2005) found that shared environmental influences play a significant role in rule-breaking behaviors (delinquency). Other studies have also found a substantial genetic component (around 65 %), but no significant shared environmental component for aggression, whereas delinquent behavior has shown a moderate genetic component (around 35 %) and a moderate shared environmental component (around 35 %; e.g., Edelbrock et al. 1995; Eley et al. 2003). Further, aggressive behavior has been shown to be more stable over time compared to delinquent behavior: after about age 10, aggressive behavior declines, whereas delinquent behavior increases until about age 17 (Stanger et al. 1997). Additionally, aggressive adjudicated youth showed greater deficits in executive neuropsychological functions (such as reasoning, problem solving and planning) than nonaggressive adjudicated youth (Moffitt and Henry 1989).

Aggressive behavior could also be divided in several subtypes on the basis of different developmental trajectories, antecedents, and consequences. First, aggression incorporates not only the infliction of physical harm, but also consists of more subtle forms of aggressive behavior, such as social exclusion. These subtle forms of aggression are referred to as indirect aggression, relational aggression, and social aggression (Vitaro et al. 2006). The different developmental trajectories of different types of aggressive behavior are demonstrated by the overtness-covertness dimension of antisocial behavior (Loeber and Schmaling 1985). The covert pathway (indirect aggression) consists of hostility, irritability, suspicion and anger, whereas the overt pathway (direct aggression) consists of verbal or physical aggression, such as fighting (Lange et al. 1995).

## The Present Study

In summary, previous research has shown that externalizing behavior of adolescents can be explained by the presence of cognitive distortions, low levels of self-esteem, low degree of parental monitoring and affiliations with deviant peers, which in turn can be explained by poor attachment quality. However, to our knowledge, previous studies have not examined whether the association between attachment and externalizing behavior is mediated by any of these factors (simultaneously). Only Simons et al. (2001) and Gomez and McLaren (2007) found that levels of self-esteem mediated the relation between attachment and aggressive behavior of adolescents. In the present study, we will not only examine mediating effects of individual mechanisms (self-esteem and cognitive distortions), but also of social mechanisms (i.e., affiliation with peers and parental monitoring).

Given that there are distinct patterns of antisocial behavior (multidimensional construct), we differentiate between direct and indirect aggression and delinquent behavior. Based on earlier research (e.g., Tackett et al. 2005), we expect that social mechanisms, considered as ‘environment influences’, play a more important role in the relation between attachment and delinquency, whereas individual mechanisms are expected to be more influential in explaining the relation between attachment and aggressive behavior. Therefore, we will examine two separate mediation models for delinquency and aggression.

## Method

### Participants and Procedure

Data were obtained from adolescents who were referred to a youth care organization and enrolled in programs for youth at risk for criminal behavior and youth who had committed minor delinquent acts in the period of 2011–2013. Participation in these programs was voluntary. Treatment professionals (specialized in child psychology) determined whether adolescents were eligible for participation on the basis of following criteria: age 12 to 23 years, experiencing problems in multiple life domains (school, family, peers, leisure time), and at risk for the development and progression of a deviant life style. For example, predelinquents with antisocial behavior, first time offenders, and adolescents with mainly minor (first) police contacts and offenses (such as, inflicting damage or destroying property on purpose, shoplifting and joyriding) were eligible for participation. Juveniles with a longer history of delinquent acts or showing severe psychopathology before age 12 were excluded from participation. After screening (for eligibility) and consent to participate, adolescents were asked to complete a questionnaire. The Ethics Committee of the University of Amsterdam (2011-CDE-01) approved the study design, procedures and informed consent.

A total of 160 adolescents were eligible and approached for participation. Finally, data of the first measurement on demographics, parental attachment and externalizing behavior were available for 102 adolescents (63.8 %). 36.2 % (*n* = 58) of the included adolescents declined to participate on the first measurement because of several reasons (8 parents and 20 juveniles did not consent to participate; 15 juveniles could not be reached; 15 other reasons, such as migration/language problems). Results of independent t-tests and chi-square tests showed no differences between participants and non-participants in age, ethnic background and gender.

All participants, aged 12 to 19 years, lived in the urban area of Amsterdam (the Netherlands). The sample of participants mainly consisted of the major ethnic groups in Dutch large cities: native Dutch (*n* = 20; 20 %), Moroccan (*n* = 26, 26 %), Turkish (*n* = 9; 9 %), and Surinamese (*n* = 26, 26 %). The remaining participants had other ethnic backgrounds (*n* = 21; 21 %). Ethnic group membership was defined by the birth country of both parents and the adolescent (native Dutch: if both parents were born in the Netherlands). About 34 % of the participants reported living with both parents, 53 % reported living with one parent (mother or father), 3 % reported living partly with both parents, and 10 % reported living with other relatives. Additional characteristics of the total sample are presented in Table 1.

**Table 1:** Sample characteristics for the total sample (*N* = 102)

	M	SD
Age	15.52	1.53
Gender (Male)	72^a^	70.6^b^
Cognitive distortions	2.61	0.68
Parental monitoring	2.89	0.62
Self-esteem	3.07	0.62
Deviant peers	1.64	0.72
Direct aggression	0.61	0.23
Indirect aggression	0.45	0.22
Delinquency	4.24	4.45
Trust (attachment)	3.13	0.81
Communication (attachment)	2.78	0.85
Alienation (attachment)	3.26	0.63

Attachment = Trust, Communication and Alienation

^a^ *n*

^b^%

### Measures

#### Parental Attachment

The attachment relationship between the adolescent and parent was assessed using the short version of the ‘Inventory of Parent and Peer Attachments’ (IPPA; Armsden and Greenberg 1987). This instrument was designed to assess the extent to which adolescents felt secure by measuring the adolescents’ trust in availability and sensitivity of the attachment figure, the quality of communication and the extent of anger and alienation in the relationship with the attachment figure. The IPPA is a 12-item self-report questionnaire using a 4-point Likert-type scale (1 = almost never, to 4 = almost always). Examples of statements for each scale are: “If my parent knows something is bothering me, he/she asks me” (communication); “My parent respects my feelings” (trust); “I don’t get much attention from my parent” (alienation). The IPPA proved to be reliable and valid in previous studies (Armsden and Greenberg 1987; Deković and Meeus 1997; Raja et al. 1992). Based on the dissatisfactory outcomes of reliability analyses and low item-total correlations on the subscales of communication (α = 0.53) and trust (α = 0.32), two items were deleted (communication scale: “my parents have their own problems, so I don’t bother them with mine”; trust scale: “I wish I had different parents”), which resulted in Cronbach’s alphas of respectively 0.74, 0.76. Cronbach’s alpha of the alienation scale was 0.62. Higher scores indicated more attachment security.

#### Cognitive Distortions

The ‘How I Think Questionnaire’ (HIT, Barriga and Gibbs 1996) was used to assess cognitive distortions of adolescents. The HIT is based upon Gibbs and colleagues’ four-category typology of self-serving cognitive distortions: self-centered attitude; blaming others; minimizing-mislabeling (consequences of) behavior; and assuming the worst (Barriga et al. 2000; Gibbs et al. 1995, 2001). For the present study we used the Dutch validated version of the HIT (Nas et al. 2005). The HIT is composed of 54 items, 39 represent the four types of self-serving cognitive distortions, 8 items are used to screen for anomalous responses, and 7 items are positive filler items. Participants responded on a 6-point scale ranging from agree strongly to disagree strongly. Examples of statements for each subscale are: “If someone is careless enough to lose a wallet, they deserve to have it stolen (self-centered); People force me to lie when they ask too many questions (blaming others); Everybody breaks the law, it’s not a big deal (minimizing); You should hurt people first, before they hurt you (assuming the worst).” Scores were averaged across items. In the present study, a total mean score of the four types of self-serving cognitive distortions items was used (39 items). Previous research documented good test-retest reliability for the HIT as well as evidence for construct validity (as described in Barriga et al. 2008). Cronbach’s alpha in the present study was found to be 0.91. Higher scores indicated more cognitive distortions.

#### Self-Esteem

Feelings of worth and satisfaction with self were measured by using the ‘Competentie Belevingsschaal voor Adolescenten’ (CBSA; Treffers et al. 2002). This questionnaire is a Dutch version of the five-item global self-worth subscale from the ‘Self-Perception Profile for Adolescents’ (SPPA, Harter 1988). Adolescents first chose which of two descriptions described them better (e.g., “Some youngsters are often disappointed in themselves”; “Other youngsters are almost never disappointed in themselves”), then they reported whether that description was a little true or totally true for them. Scores were averaged across items. Higher scores indicated a greater sense of self-worth. The internal consistency of the scale of global self-worth was found to be good, α = 0.80 (Evers et al. 2007). The present study’s reliability analysis resulted in a satisfactory Cronbach’s alpha of 0.64.

#### Parental Monitoring

Parental knowledge of adolescents’ whereabouts was measured by the ‘Vragenlijst Toezicht Houden’ (VTH; Deković 1996), the Dutch version of the five-item parental monitoring scale used in previous studies (e.g., Brown et al. 1993). Adolescents responded on a 4-point Likert-type scale (1 = nothing, 2 = a little, 3 = a lot, 4 = everything) how much their parents know about who their friends are; how they spent their money; where they were after school; which place they went when they left home; what they did in their leisure time; and what grades they received at school. Scores were averaged across items. Higher scores indicated more parental monitoring. Brown et al. (1993) found an acceptable internal consistency of five-item scale of parental monitoring (α = 0.80). The good internal consistency of the scale of parental monitoring was confirmed in present study, α = 0.84.

#### Deviant Peer Affiliation

Adolescents’ perceptions of deviant peer affiliation were measured by the Dutch version of the ‘Family, Friends and Self Scale’ (‘Delinquentie van Leeftijdgenoten’, Deković 1999; Deković et al. 2004) of Simpson and McBride (1992). Adolescents indicated on 10 items how many of their friends participated in a variety of deviant behaviors (e.g., purposely damage or destroy property) on a scale from 1 (none of my friends) to 5 (almost all of my friends). Scores were averaged across items. Higher scores indicated a higher number of deviant friends. The good internal consistency of the FFS scale was proved by Simpson and McBride (1992). The internal consistency of the scale in present study was found to be excellent, α = 0.91.

#### Aggressive Behavior

The ‘Buss-Durkee Hostility Inventory’ (BDHI), developed by Buss and Durkee (1957), was used to measure adolescents’ aggression. The BDHI consists of two subscales ‘Direct Aggression’ (measuring the tendency to express verbal or physical aggression) and ‘Indirect Aggression’ (determining the emotional and cognitive components: hostility, irritability, suspicion, and anger). Results of the present study are based on the two scales of direct and indirect aggression of the Dutch validated 35-item version of the Buss-Durkee Hostility Inventory (BDHI-D) of Lange et al. (1995). The good internal consistency of the BDHI subscales was proved by previous research (Lange et al. 1995). Cronbach’s alphas of the subscales ‘direct’ and ‘indirect’ aggression in present study were both 0.78 (α = 0.85 total scale). Each item was rated as 0 (not true) or 1 (true). Scores were averaged across items. Higher scores indicated higher levels of aggressive behavior.

#### Delinquent Behavior

Participation and versatility in delinquency were assessed by the ‘Self-report Delinquency Scale’ (SRD, Van der Laan and Blom 2006). Participants responded on 33 items if they participated in diverse delinquent acts, based on six subscales: property damage, property and theft, violent acts, weapon possession, drugs possession and dealing, and cybercrime. Sum scores of participation in 33 delinquent acts were used for the analyses, with higher scores indicating more delinquent behavior. Cronbach’s alpha was 0.86.

### Analytic Strategy

**Fig. 1:**
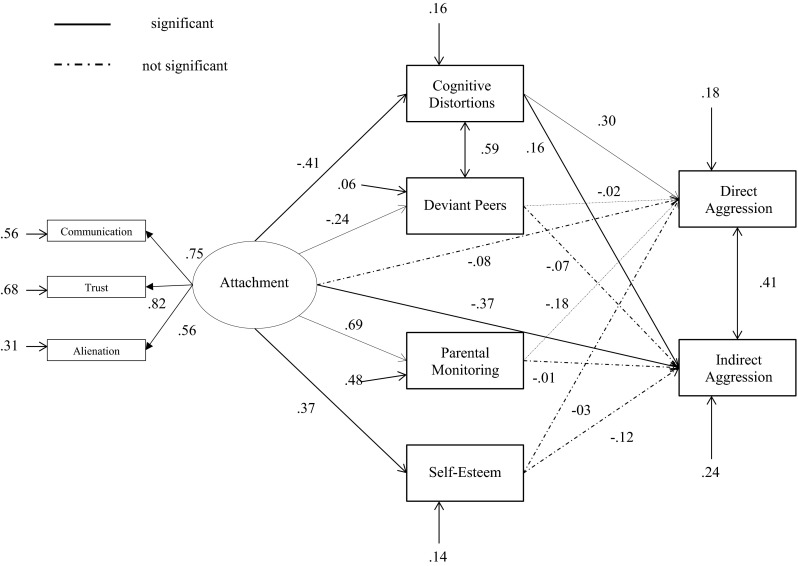
Structural equation model with standardized parameters estimates: direct and indirect aggression

**Fig. 2:**
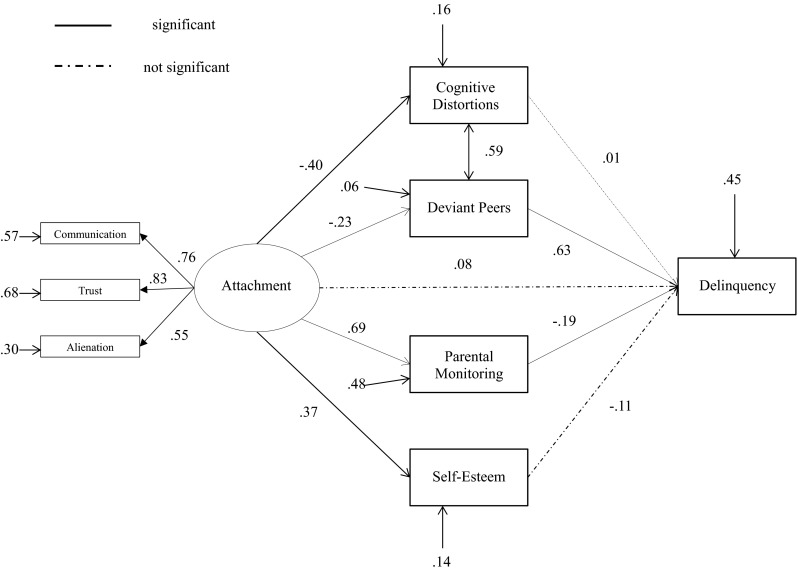
Structural equation model with standardized parameters estimates: delinquency

First, bivariate correlation analyses were conducted between all study variables of the total sample. Next, we tested two separate models for direct and indirect aggression (see Fig. 1) and delinquency (see Fig. 2). The mediating paths of the relation between attachment and (direct/indirect) aggression and between attachment and delinquency were evaluated using structural equation modeling (SEM) techniques. The software package Mplus (Muthén and Muthén 2007) was used to fit the proposed model to the data. Delinquency, cognitive distortions, parental monitoring, deviant peers, self-esteem, and attachment were treated as censored variables. Censored variables are variables with a large fraction of observations at the minimum or maximum value. Many respondents had lower scores on delinquency, deviant peers, cognitive distortions and higher scores on parental monitoring, self-esteem and attachment. The regression coefficients of the censored dependent variables are described as ‘Tobit regression coefficients’ (Tobin 1958). By means of Mplus models with categorical and censored variables with ‘Weighted Least Squares Mean and Variance’ (WLSMV) can be tested.

The assessment of SEM models and evaluation of the fit of the models is based on the chi-square (*χ* ^2^), the corresponding *p*-value, the comparative fit index (CFI, Bentler 1990), and the root mean squared error of approximation (RMSEA, Steiger and Lind 1980). Good-fitting models show a non-significant *χ* ^2^. Values of the RMSEA less than 0.05 are considered to indicate a good fit, with values between 0.05 and 0.08 indicating a fair fit. Values of CFI above 0.90 are generally regarded as evidence for a good fit (Hu and Bentler 1999; Hox and Bechger 1998). Modifications indices (MI’s) were used to guide model specification and improvement of the CFI value (>0.95) or RMSEA value (<0.05). The chi-square difference test, the DIFFTEST-option in Mplus, was used to assess the difference in fit between the hypothesized and alternative model. Finally, an alpha level of *p* < 0.05 (two-tailed) was used for significance and *p* < 0.10 was used to report trends.

## Results

Preliminary bivariate correlation analyses revealed significant associations between the variables for the total sample (results are available as supplementary online material). A structural equation model was used to examine the association between adolescent-parent attachment and delinquent and direct and indirect aggressive behavior of adolescents. The modeling procedure started by fitting a mediation model with paths from attachment to the mediating factors and from the mediating factors to delinquent and aggressive behavior. Next, the fit of the model for the total sample was evaluated. We expected that the IPPA subscales (communication, trust, and alienation) would form a latent factor for adolescents’ attachment to parents and thus allow for a more parsimonious model.

Given that we found no significant correlations between age and gender and three dimensions of attachment, we decided not to include age and gender as covariates in the model. The first mediation model of adolescent-parent attachment and direct and indirect aggression did not provide an acceptable fit to the data, *χ* ^2^ (18, *N* = 102) = 52.23, *p* < 0.001, CFI = 0.866, RMSEA = 0.137. After examination of the modification indices, we added the relation between deviant peers and cognitive distortions. This significantly improved the fit of the model, Δχ^2^(1) = 31.08, *p* < 0.001, and the final model provided a close and acceptable fit to the data, *χ* ^2^ (17, *N* = 102) = 23.33, *p* > 0.05, CFI = 0.975, RMSEA = 0.060. The fit statistics for the resultant model of attachment and direct and indirect aggression are presented in Fig. 1.

A similar procedure was used for the mediation model of adolescent-parent attachment and delinquency. The first mediation model of parental attachment and delinquency did not provide an acceptable fit to the data, *χ* ^2^ (16, *N* = 102) = 49.92, *p* < 0.001, CFI = 0.876, RMSEA = 0.144. After examination of the modification indices, we added the relation between deviant peers and cognitive distortions. This significantly improved the fit of the model, Δχ^2^(1) = 31.76, *p* < 0.001, and the final model showed a close and acceptable fit to the data, *χ* ^2^ (15, *N* = 102) = 22.84, *p* > 0.05, CFI = 0.971, RMSEA = 0.072. The fit statistics for the resultant model of attachment and delinquency are presented in Fig. 2.

Tests of indirect effects revealed full mediation from adolescent-parent attachment to direct aggression via adolescents’ cognitive distortions (ß = −0.12, *p* < 0.01) and partial mediation from parental attachment to indirect aggression via adolescents’ cognitive distortions (ß = −0.07, *p* < 0.05), the independent variable had still a significant effect on the dependent variable (*p* < 0.01). Furthermore, the results revealed that parental attachment was indirectly related to delinquency via adolescents’ deviant peers (ß = −0.15, *p* < 0.001). A trend was found regarding the indirect path via parental monitoring (ß = −0.13, *p* = 0.09). No indirect effects were found for parental monitoring, self-esteem and deviant peers as mediators of the relation between attachment and direct and indirect aggression. With regard to the relation between attachment and delinquency, we found no indirect effects of cognitive distortions and self-esteem. Table 2 presents the estimates of the standardized indirect effects.

**Table 2:** Standardized indirect effects from attachment to aggression and attachment to delinquency

Indirect effect via	Direct aggression	Indirect aggression	Delinquency
Cognitive distortions	−0.12 (0.05)**	−0.07 (0.03)*	−0.00 (0.05)
Deviant friends	0.01 (0.02)	0.02 (0.02)	−0.15 (0.04)***
Parental monitoring	−0.13 (0.09)	−0.00 (0.08)	−0.13 (0.08)+
Self-esteem	0.01 (0.04)	−0.05 (0.04)	−0.04 (0.04)

Standard errors in parentheses

+ *p* < 0.10; * *p* < 0.05; ** *p* < 0.01; *** *p* < 0.001 (two-tailed)

## Discussion

Although attachment insecurity has been found to be related to externalizing problem behavior, the possible mechanisms underlying this relation have, to our knowledge, never been empirically tested. Therefore, the aim of the present study was to examine the association between adolescent-parent attachment and externalizing behavior of adolescents, and whether this association was mediated by cognitive distortions, self-esteem, parental monitoring, and deviant friends. We distinguished between delinquent and aggressive behavior, because these types of externalizing behavior represent two distinct clinical concepts, and are characterized by different developmental trajectories (Stanger et al. 1997).

As expected, the present results revealed that the relation between poor attachment and (direct and indirect) aggression was mediated by individual factors (adolescents’ cognitive distortions), whereas the association between attachment and delinquent behavior was mediated by social factors, such as affiliations with deviant peers and parental monitoring. Contrary to our expectations, the hypothesized mediating role of self-esteem in the relation between attachment and aggression was not supported.

We found full mediation for direct aggression, suggesting that cognitive distortions play a significant role in the relation between attachment and direct aggression. Only partial mediation was found for indirect aggression. Internal working models of attachment contribute to the way adolescents view others (Bowlby 1969), which in particular may be related to the indirect and covert subtype of aggression. This type of aggressive behavior involves social manipulation of peer relationships in order to harm another individual (Vitaro et al. 2006). In this respect, other aspects of adolescents’ cognitions may play a role in mediating the association between attachment and indirect aggression, too. For example, Capuano (2011) found that the interaction between cognitive distortions and the perspective-taking component of empathy predicted indirect (social) aggression, whereas direct (physical) aggression was only predicted by cognitive distortions. These findings confirm that indirect and direct aggression show specific developmental trajectories, which are characterized by the overtness-covertness dimension of antisocial behavior (Loeber and Schmaling 1985).

In contrast with findings of Simons et al. (2001) and Gomez and McLaren (2007), self-esteem proved not to be a significant mediator of the associations between attachment and both types of aggressive behavior. We found no significant relation between self-esteem and direct aggression, and a relatively weak relation between self-esteem and indirect aggressive behavior. Findings of previous research on the link between self-esteem and externalizing problems are equivocal. Although several researchers argued that levels of self-esteem are related to externalizing problems (e.g., Donnellan et al. 2005; Fergusson and Horwood 2002), others have questioned this claim (Jang and Thornberry 1998; Matsueda 1992; Rosenberg et al. 1995). For example, Rosenberg et al. (1995) showed that content-specific self-concept (such as academic self-esteem) is more strongly related to behavioral outcomes, whereas global self-esteem is associated with psychosocial well-being. Thus, the present results could be explained by the way in which self-esteem was measured.

Findings of the present study confirm that social factors, namely affiliation with deviant peers and low parental monitoring, play a more important role in mediating the association between poor attachment bonds and delinquency, than the relation between attachment and aggressive behavior. The present study includes adolescents who may be characterized as adolescent-onset delinquents (Moffitt 1993; Patterson and Yoerger 1997), as juveniles were enrolled in the treatment program on the basis of first police contact and absence of a longer history of delinquent acts or severe psychopathology. Adolescent-onset delinquency is thought to be mainly predicted by societal and environmental factors. It is assumed that adolescent-onset delinquents experience a maturity gap (Moffitt 1993), characterized by a dramatic shift in self-perceptions of autonomy and self-reliance. When experiencing discomfort with the maturity gap, adolescents enter a social reference group at high school. This reference group is characterized by peers who have already been involved in delinquent ways of coping with the maturity gap (Moffitt 1993). The deviant peer group forms a key role in training covert antisocial and delinquent behaviors among adolescent-onset delinquents. Patterson and Yoerger (1997) emphasized the negative influence of deviant peers as the mediating mechanism between family process and late-onset arrest, which is consistent with our findings.

### Study Limitations

There are several limitations of this study that should be noted. First, the cross-sectional nature of this study precludes a causal interpretation of the results. Therefore, longitudinal research is needed to gain more insight in the mediation patterns implied by the current study. Second, data from present study were derived from a sample participating in a randomized controlled trial (RCT). Unfortunately, selection is a common methodological problem in experimental (RCT) designs (Asscher et al. 2007; Farrington and Welsh 2005). A possible selection bias (the possibility that the more severely affected adolescents may have declined participation) cannot be ruled out in present study. However, we found no pre-existing differences between participants and non-participants on demographic factors based on attrition analyses. Third, our study is only based on self-reports of adolescents, which increases the chance of overestimating the strength of association due to method variance. Therefore, further research should involve multiple informants (parents, siblings and teachers) when examining underlying mechanisms of the association between attachment and externalizing behavior.

Fourth, although the Inventory of Parent and Peer Attachment (IPPA) is considered to be a reliable self-report instrument of adolescent-parent attachment, it cannot distinguish between qualitatively different patterns of attachment, and does not assess internal working models of attachment (Lyddon et al. 1993). Notably, only a limited number of validated self-report questionnaires measuring attachment styles for pre, middle and late adolescents are available (Jones et al. 2014). These questionnaires address different aspects of attachment compared to in-depth interviews, which primarily assess attachment representations (Jones et al. 2014; Ravitz et al. 2010). Future research should use valid instruments (which need to be developed) measuring both adolescents’ mental representations of attachment and attachment styles in order to fully capture the relation between attachment and externalizing behavior in adolescents.

Fifth, we did not evaluate attachment of adolescents towards mothers and fathers separately. Mother- and father relationships with the adolescent may be differentially predictive of certain developmental outcomes (Rice 1990). For example, Grossmann et al. (2002) showed that children’s model of the self as competent and worthy of help derives from different experiences with the father and mother as attachment figures. Further exploration of these specific relationships would be interesting in future studies. Sixth, it is important to stress that cognitive distortions, parental monitoring and deviant peers only partly mediate the association between attachment and externalizing behavior problems. Further research should explore additional underlying social and individual mechanisms that may explain the relation between secure attachment bonds and risk for externalizing behavior, such as the capacity of effective emotion regulation (e.g., Cassidy 1994) and the socialization of moral emotions and values within a secure relationship (Kochanska 1997; Van IJzendoorn 1997).

Finally, we did not examine the mediation patterns for different subgroups, such as boys and girls, and at different ages. The small sample size of the present study restricted conducting a multiple group analysis (for gender- and age groups). However, Hoeve et al. (2012) found that poor bonds to parents similarly explain delinquency in boys and girls. With regard to the role of age, the association between attachment and externalizing behavior seems to depend on important developmental periods of youngsters (in the transition to adolescence, see Rice 1990). A longitudinal research design, based on a more heterogeneous and larger sample is needed to test mediation models of attachment and problem behavior for different phases in childhood and (pre-, middle, and late) adolescence.

Further research should also be conducted for examination of the hypotheses of the current study in different populations, including groups at the extremes of adolescent-parent attachment and externalizing behavior. In the present sample, adolescents tended to report relatively secure attachment relationships with their parent, and there were very few reports of high levels of delinquent behavior.

### Implications for Clinical Practice and Research

The findings of the present study imply that prevention and treatment of aggressive and delinquent behavior should not neglect links between attachment to parents and peer relationships, parental monitoring through adolescents’ self-disclosure and cognitive distortions. Consequently, improvement of adolescent-parent attachment bonds may be expected to diminish aggressive behavior since this could reduce cognitive distortions that may, in turn, reduce aggression. In addition, focusing on the attachment patterns between adolescents and their parents may solve problems related to deviant friendships and low levels of parental monitoring, which in turn could reduce adolescents’ involvement in delinquent activities.

Several meta-analytic studies of preventive and curative interventions showed that involving the family system leads to reductions in conduct problems of adolescents (De Vries et al. 2014; Farrington and Welsh 2003; Litschge et al. 2010; McCord et al. 2001; Van der Stouwe et al. 2014). Improvement of the attachment relationship between parents and adolescents could be one of the targets within these family-based programs.

## Electronic supplementary material

Below is the link to the electronic supplementary material.

ESM 1 (DOCX 17 kb)
