# Supplementary material for: Adolescent-Parent Attachment and Externalizing Behavior: The Mediating Role of Individual and Social Factors
Source: J Abnorm Child Psychol. 2015 Mar 15;44(2):283–94. doi: 10.1007/s10802-015-9999-5 (PMC4729816; doi:10.1007/s10802-015-9999-5)
Supplement: Supplementary file 1 — (DOCX 17 kb) [file 10802_2015_9999_MOESM1_ESM.docx]

*Correlations between Demographic Characteristics, Parental Attachment, and Externalizing Behavior*

|  | Variable | 1 | 2 | 3 | 4 | 5 | 6 | 7 | 8 | 9 | 10 |
| --- | --- | --- | --- | --- | --- | --- | --- | --- | --- | --- | --- |
| 1 | Cognitive Distortions | - |  |  |  |  |  |  |  |  |  |
| 2 | Parental monitoring | -.31** | - |  |  |  |  |  |  |  |  |
| 3 | Self-Esteem | -.29** | .12 | - |  |  |  |  |  |  |  |
| 4 | Deviant Peers | .51** | -.23* | -.16 | - |  |  |  |  |  |  |
| 5 | Direct Aggression | .37** | -.30** | -.06 | .27** | - |  |  |  |  |  |
| 6 | Indirect Aggression | .31** | -.30** | -.28** | .17 | .48** | - |  |  |  |  |
| 7 | Delinquency | .33** | -.28** | -.16+ | .59** | .35** | .22* | - |  |  |  |
| 8 | Trust | -.42** | .52** | .21* | -.20* | -.24* | -.32** | -.07 | - |  |  |
| 9 | Communication | -.36** | .57** | .25* | -.14 | -.21* | -.31** | -.21* | .63** | - |  |
| 10 | Alienation | -.27** | .28** | .26** | -.10 | -.23* | -.36** | -.21* | .34** | .31** | - |

*Note*. *N* = 102. Attachment = Trust, Communication and Alienation. Externalizing Behavior = Delinquency, Direct and Indirect Aggression.

+ *p* < .10, * *p* < .05; ** *p* < .01 (two-tailed)
